# Murine Typhus: An Important Consideration for the Nonspecific Febrile Illness

**DOI:** 10.1155/2012/134601

**Published:** 2012-12-24

**Authors:** Gurjot Basra, Megan A. Berman, Lucas S. Blanton

**Affiliations:** ^1^Division of General Medicine, Department of Internal Medicine, The University of Texas Medical Branch, 301 University Boulevard, Galveston, TX 77555, USA; ^2^Division of Infectious Diseases, Department of Internal Medicine, The University of Texas Medical Branch, 301 University Boulevard, Galveston, TX 77555, USA

## Abstract

Murine typhus is a widely distributed flea-borne infection caused by *Rickettsia typhi*. Symptoms of murine typhus are nonspecific and mimic a variety of other infectious diseases. We herein report a case of murine typhus in an area where the broad use of DDT in the mid-20th century has now made it a rare disease. The patient described presented with headache, fever, and a faint macular rash. Initial laboratory studies revealed a slight transaminase elevation. Further questioning revealed exposure to opossums, prompting the consideration of murine typhus as a diagnosis. Although typhus group antibodies were not present during the patient's acute illness, empiric therapy with doxycycline was initiated, and the patient defervesced. One month after convalescence, the patient returned to clinic with serum that contained typhus group antibodies with an IgG titer of 1 : 1024. Murine typhus is an important consideration during the workup of a patient with a nonspecific febrile illness. Exposure to reservoir hosts and the flea vector place humans at risk for this disease. Clinician recognition of this entity is required for diagnosis and effective therapy.

## 1. Introduction

Murine typhus is a flea-borne infection caused by *Rickettsia typhi*. It is widely distributed and endemic to warm coastal areas throughout the world. Symptoms of murine typhus are relatively nonspecific and mimic a variety of other infectious diseases. Diagnosis usually requires convalescent antibody testing. This situation makes a definitive diagnosis during the acute stage of illness difficult to establish [1]. Although control of the flea vectors can effectively control the burden of human disease [2, 3], the peridomestic invasion of reservoir hosts (e.g., rats and opossums) places people at risk for disease [4–6]. We herein report a case of murine typhus in an area where the broad use of DDT in the mid-20th century made it a rare disease [2, 3].

## 2. Case Report

A 59-year-old Hispanic male from Galveston, Texas presented to the Emergency Department on April 29, 2012 with complaints of headache and fever. His headache started two weeks prior, and he noted temperatures up to 40 degrees Celsius in the preceding five days. He denied neck pain, visual complaints, respiratory symptoms, or arthralgias. He denied recent travel, eating unpasteurized milk products, and sexual activity for the last several years. Two days prior to presentation, he was evaluated by the Emergency Department and was discharged with a presumed viral illness. Because of persistent fever and headache, he returned to the Emergency Department where he was admitted for further evaluation. 

His medical history was significant for open-angle glaucoma and seasonal allergies. Outpatient medications included travoprost and dorzolamide ophthalmic drops. Multiple family members had diabetes mellitus and hypertension, and his father had a diagnosis of leukemia. He denied use of tobacco or illicit drugs. He drank alcohol rarely. 

**Figure 1:**
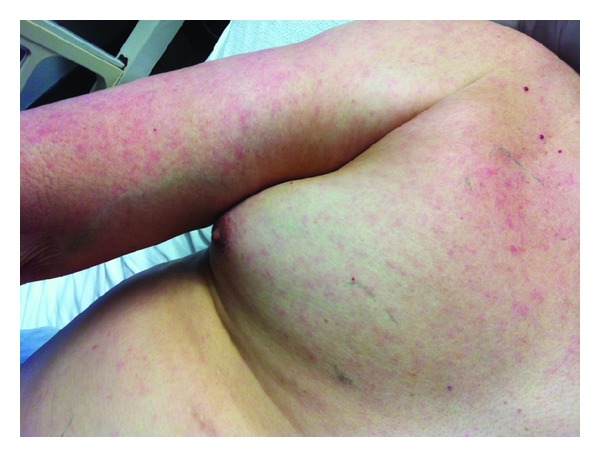
A light erythematous macular rash was noted on the chest and upper extremities.

On examination, his temperature was 39.2 degrees Celsius, blood pressure 117/78 mmHg, heart rate 75 beats/minute, respiratory rate 16 breaths/minute, and oxygen saturation 95% on ambient air. He appeared ill, and skin examination was remarkable for a lightly erythematous macular rash on his chest and arms (Figure 1). The examination was otherwise unremarkable.

Laboratory data revealed a leukocyte count of 3700/*μ*L (3500 to 10,000/*μ*L), erythrocyte sedimentation rate of 15 mm/hour (0 to 10 mm/hour), C-reactive protein of 12.3 mg/dL (0.0 to 0.8 mg/dL), ferritin of 1320 ng/mL (18 to 464 ng/mL), aspartate aminotransferase of 66 units/L (13 to 40 units/L), and alanine transaminase of 94 units/L (9 to 51 units/L). Serologic tests for viral hepatitis, HIV, cytomegalovirus, Epstein-Barr virus, syphilis, and *Bartonella* were nonreactive. Anti-nuclear antibody, antibodies to double-stranded DNA, and rheumatoid factor were negative. Blood and urine cultures were negative. Cerebrospinal fluid analysis was unremarkable. Chest radiograph and computed tomography of the head, abdomen, and pelvis were negative for lesions suggestive of malignancy or focal infectious process. 

Throughout hospitalization, the patient continued to have daily fevers with temperatures up to 40 degrees Celsius. A careful revisitation of his history revealed exposure to opossums around his yard and home. This historical information in combination of his symptoms, rash, and mild transaminitis rendered murine typhus a likely diagnosis. Doxycycline was initiated and was followed by complete resolution of his symptoms within 48 hours. Although an immunofluorescence IgG assay for typhus group antibodies was nonreactive, the patient was discharged to complete a seven-day course of doxycycline. 

One month after discharge, the patient returned to clinic and was free of symptoms. Serum obtained for an immunofluorescence IgG assay for typhus group antibodies was reactive at a dilution of 1 : 1024, confirming the diagnosis of murine typhus. 

## 3. Discussion

Murine typhus is caused by *Rickettsia typhi*, an obligately intracellular gram-negative bacterium. The agent is transmitted by inoculation of infected flea feces into flea bites and targets human endothelial cells. In subtropical and tropical coastal areas, rats act as reservoirs, and the disease is transmitted by the bite of the oriental rat flea (*Xenopsylla cheopis*) [1]. In the United States, urban and suburban transmissions via the cat flea (*Ctenocephalides felis*) have been linked to the opossum as a reservoir host [4, 5]. Another rickettsial organism, *Rickettsia felis*, shares the same zoonotic hosts and flea vectors as *R. typhi* [7] but differs phylogenetically and shares slight and variable cross-reactivity with organisms belonging to the typhus group [8]. 

The most common signs and symptoms of murine typhus are nonspecific and include fever (98%–100%), headache (75%–77%), and chills (66%). Rash is relatively common (54%–63%) and may be the clue that raises suspicion for murine typhus [9–11]. The rash is usually macular but may also be maculopapular, papular, petechial, or morbilliform [9]. History of flea exposure and preceding flea bites is typically not recalled [1, 9, 10]. Laboratory abnormalities may include leukopenia and moderately elevated hepatic transaminases [1].

Diagnosis can be made by antibiotic free cell culture, immunohistochemical analysis of tissues, or polymerase chain reaction amplification of rickettsial DNA in blood. These techniques require specialized laboratories and techniques not readily available to most clinicians. Therefore, the diagnostic tests of choice are serologic methods to demonstrate a fourfold or greater rise from acute to convalescent antibody titers of IgG specific to typhus group antigen. As in the case presented, typhus group antibodies may not be present in the first week of illness. Antibodies are present in almost all patients within two weeks [1]. Although a slight yet variable serologic cross-reaction can occur with other rickettsiae (i.e., *R. felis*) [8], the strong reaction to typhus group antigen supports *R. typhi* as the cause of this patient's illnesses. Since serologic diagnosis is retrospective, prompt empiric therapy with a tetracycline is imperative when murine typhus is suspected. Although death is uncommon (case fatality 1% to 4%), timely treatment can decrease the duration and severity of symptoms [1].

Murine typhus was once a common disease in the Gulf Coast city of Galveston, Texas. Efforts to decrease the burden of rat ectoparasites by intensive dusting with DDT virtually eliminated this disease [2, 3]. Although the southern counties of Texas and California currently report the greatest number of murine typhus cases in the United States, the disease is still uncommonly diagnosed. It is likely vastly underdiagnosed and mistaken for other febrile illnesses [7]. A large outbreak of murine typhus in Austin, Texas (an area of historically rare occurrence) demonstrates the susceptibility of a population when in close proximity to animal reservoirs such as cat flea-infested opossums [6]. 

Awareness of uncommon, yet endemic, pathogens in a physician's area of practice is important for the timely diagnosis and effective therapy of any nonspecific febrile illnesses. Murine typhus exemplifies this principle. In summary, the aforementioned case report illustrates several key issues when considering murine typhus as a cause of fever. The diagnosis must be considered in areas where the reservoir hosts are in close contact with humans. Definitive diagnosis is usually retrospective (requiring convalescent antibody titers). Finally, empiric treatment should not be withheld while awaiting diagnostic studies.
